# The effects of mirror therapy with neuromuscular electrical stimulation on motor and sensory functions in patients with common peroneal nerve injury

**DOI:** 10.3389/fnins.2024.1486959

**Published:** 2025-01-13

**Authors:** Xiaolei Chu, Jiajia Liang, Mingwei Gao, Xiaoxuan Zhao, Jiaojiao Sun, Wenjie Liu, Donglin Zhao, Zheng Xing, Qi Li

**Affiliations:** ^1^Department of Rehabilitation, Tianjin Hospital, Tianjin, China; ^2^Tianjin Key Laboratory of Exercise Physiology and Sports Medicine, Institute of Sport, Exercise and Health, Tianjin University of Sport, Tianjin, China

**Keywords:** neuromuscular electrical stimulation, mirror therapy, common peroneal nerve, peripheral nerves, motor function, sensory function

## Abstract

**Background:**

Injuries to the common peroneal nerve often result in significant sensory and motor function loss, severely affecting patients’ quality of life. Although existing treatments, including medication and surgery, provide some degree of efficacy, their effectiveness is limited by factors such as tolerance and adverse side effects.

**Methods:**

This study aims to evaluate the effects of a 4-week regimen of mirror therapy combined with neuromuscular electrical stimulation on lower limb function, muscle strength, and sensation in patients with common peroneal nerve injuries. The objective is to identify novel therapeutic strategies for lower limb peripheral nerve injuries.30 patients with Common peroneal nerve caused by pelvic fractures were selected from the Rehabilitation Medicine Department of Tianjin Hospital between July 2023 and July 2024. They were randomly divided into two groups: the neuromuscular electrical stimulation group (*n* = 15) and the mirror therapy with neuromuscular electrical stimulation group (*n* = 15).

**Results:**

After 4 weeks, it was found that mirror therapy with neuromuscular electrical stimulation has a significantly better therapeutic effect on Common peroneal nerve than simple electrical stimulation therapy, particularly in terms of superficial sensation, nerve conduction velocity and ROM.

## 1 Introduction

Peripheral nerve injuries are typically caused by various factors, including physical trauma, chemical damage, metabolic disorders, or inflammation, leading to sensory and motor function loss, and potentially chronic neuropathic pain, severely affecting patients’ quality of life (Xu et al., 2021). Reports indicate that the annual incidence rate of upper limb peripheral nerve injuries in the United States averaged 36.9% between 2009 and 2018,rising to 51.9% in 2018 (Li et al., 2020). These injuries not only have a high incidence but also pose significant harm, creating a substantial economic burden on society. Apart from these factors, the recovery from peripheral nerve injury involves a series of physicochemical reactions. After aperipheral nerve injury, axonal transport is blocked, causing degeneration and disintegration of the distal part of the axon from the proximal end. Subsequently, Schwann cells proliferate massively and secrete various neuroactive substances, such as nerve growth factors and fibroblast growth factors, tore-establish connections between axons and Schwann cells, promoting nerve regeneration (Wei et al., 2023; Aszmann et al., 2015). This process is lengthy and challenging, not only due to the extended regeneration period but also the slow growth rate, with an average daily growth speed of about 2 mm. During this process, disordered axonal regeneration and erroneous reconnection with target organs can lead to pathological phenomena like hyperalgesia and sensory inversion, inhibiting further nerve regeneration (Manoukian et al., 2020).

In peripheral nerve injuries, lower limb peripheral nerve injuries are more difficult to recover from than upper limb injuries. The lower limbs are crucial support and movement organs of the human body, required for walking, jumping, running, and other complex actions, which need precise control and coordination by the nervous system. In terms of nerve structure, the nerves of the lower limbs are larger in diameter and have a longer course than those of the upper limbs, and the nerves that innervate the lower limb muscles are more complex. The lower limb nerves are thicker and more numerous because their relatively larger diameter can accommodate more nerve fibers, transmitting more nerve impulses for precise control of lower limb muscles and skin (Thatte et al., 2019). Besides structural characteristics, the challenges faced in lower limb nerve regeneration are multifaceted, including limited nerve regeneration capacity, poor regeneration environment, types of nerve damage, complications and side effects, and treatment method limitations (Murovic, 2009).Common peroneal nerve (CPN) injury is the most frequently damaged in the lower limb (Pang et al., 2024), often resulting from trauma as well as friction and compression between tissues. Given the anatomical features of the CPN, nerve damage can occur along its entire course, from its origin at the sciatic nerve to its distal branches in the foot and ankle (Poage et al., 2016). The most common site of injury is at the level of the fibular head, where compressive lesions are frequently observed. CPN injury often results in a “drop foot” symptom, with patients often exhibiting a characteristic steppage gait and suffering from ankle motor weakness in dorsiflexion. The loss of great toe extension and dorsal foot sensory is also common (Daniels et al., 2023). These impairments have a profound impact on patients’ motor function, quality of life, and mental health. Pelvic fractures are among the leading causes of common peroneal nerve injury, as they can lead to damage of the lumbosacral plexus, which in turn affects the common peroneal nerve (Chiodo, 2007; Lee and Kim, 2020). Due to the nerve’s small size, limited number of axonal fibers, and its susceptibility to compression-related damage, recovery from CPN injury presents significant challenges. Therefore, the development of effective therapeutic strategies for CPN injuries is critically important. Advances in modern medical technology, particularly in the areas of neuroelectrical stimulation, neuroprosthetics, and personalized medicine (Mela et al., 2023), have opened new pathways and approaches for the treatment of peroneal nerve injuries.

Shang et al. found that brief low-frequency electrical stimulation on the proxima lend of damaged nerves changes the potential of nerve cell membranes through external currents, rearranging charge distribution and exciting neurons, which aids in peripheral nerve regeneration (Shang et al., 2019). The primary advantage of low-frequency electrical stimulation is its ability to induce muscle contraction by stimulating motor nerves or muscles, treating related diseases Neuromuscular electrical stimulation (NMES), a type of low-frequency electrical stimulation, depolarizes nerve fibers at sufficient intensity (Senger et al., 2018). Research indicates that low-frequency pulse currents (2 Hz) can elicit single muscle contractions, characterized by a smaller cross-sectional area, a higher proportion of myelinated fibers, shorter latency, longer duration, and faster conduction speed. In contrast, high-frequency NMES can cause nerve conduction block, which, while not resulting in structural damage to the nerve, may lead to impaired nerve function. Therefore, it is evident that 2 Hz low-frequency NMES may represent a safe and effective frequency choice for the treatment of peripheral nerve injuries (Lu et al., 2008; Liao et al., 2020; Ling et al., 2019).However, pure NMES can lead to tolerance, reducing patient compliance and requiring active participation in treatment, thus a new treatment method is needed to address the above deficiencies.

Mirror therapy (MT), a new approach combining visual feedback and motor imagery, focuses on treating central nervous system disorders (Cui et al., 2022). Due to its convenience, low cost, and significant efficacy, it has become a clinical treatment method. MT functions based on the mirror neuron theory, where specific neurons in the brain a reactivated during execution, imagination, observation of an activity, or listening to activity instructions (Mollà-Casanova et al., 2024). The principle of MT is that action observation activates the motor system, inducing motor learning and improving functional remodeling. By repeatedly stimulating the brain’s mirror neuron groups through observation and execution of actions, the impaired side’s motor function can be improved. MT is primarily used for stroke rehabilitation, but recent studies have shown its potential for treating peripheral nerve injuries. Peripheral nerve injuries affect brain plasticity, causing cortical area shrinkage and signal shielding following nerve transection (Navarro et al., 2007). Incomplete postoperative functional recovery is often linked to inadequate restoration of brain signal input. MT’s advantage lies in receiving multiple sensory inputs through visual feedback and active movements, stimulating brain neurons, altering brain plasticity, and activating shielded signal input areas, thereby improving the impaired side’s function. It has been applied in forearm peripheral nerve injuries, showing better outcomes in finger and hand flexibility (Chen et al., 2022). Research on lower limb peripheral nerve injuries using MT is limited, but studies on lower limb stroke confirm its significant effects (Li et al., 2024), offering potential for further exploration.

Numerous studies have demonstrated the efficacy of NMES in treating peripheral nerve injuries (Liu et al., 2010). Early treatment with low-frequency electrical stimulation has shown significant therapeutic effects (Lee et al., 2021). However, NMES lacks active patient participation, which contradicts the principles of rehabilitation. Clinical observations show diminishing effects over time, likely due to decreased sensitivity in the muscles and nerves, as well as tolerance to prolonged stimulation. Surface electrical currents from low-frequency output have poor penetration, potentially causing local skin burns with prolonged output, leading to further patient harm (Stinear and Byblow, 2002). Given the limited efficacy of low-frequency NMES in peripheral areas, a new therapeutic approach is needed to overcome these limitations and enhance nerve recovery. Research indicates that nerve injuries often lead to signal shielding and brain area shrinkage, while mirror neurons can be activated by imagined and executed movements (Deconinck et al., 2014). By performing contralateral movements through visual observation and imagination, the affected areas can be re-stimulated, promoting bilateral activity, reducing intracortical inhibition, and improving motor function (Alahmari et al., 2020). This study aims to combine NMES with MT, where NMES provides sensory feedback from the periphery to the central nervous system, while MT controls motor functions from the central nervous system to the periphery. Together, they change brain remodeling, activate corticospinal tract pathways, and improve voluntary body movements, thereby assisting NMES to form a new treatment method, further improving the efficacy for patients with CPN injury.

## 2 Materials and methods

### 2.1 Participants

This study employed a randomized controlled trial method, including 30 patients with CPN injury due to pelvic fractures who were treated at the Rehabilitation Department of Tianjin Hospital from July 2023 to July 2024. The participants were randomly assigned to the following groups using a random function and visual binning:

1. NMES Group (*n* = 15).
2. Combined MT and NMES Group (*n* = 15).

**Figure 1:**
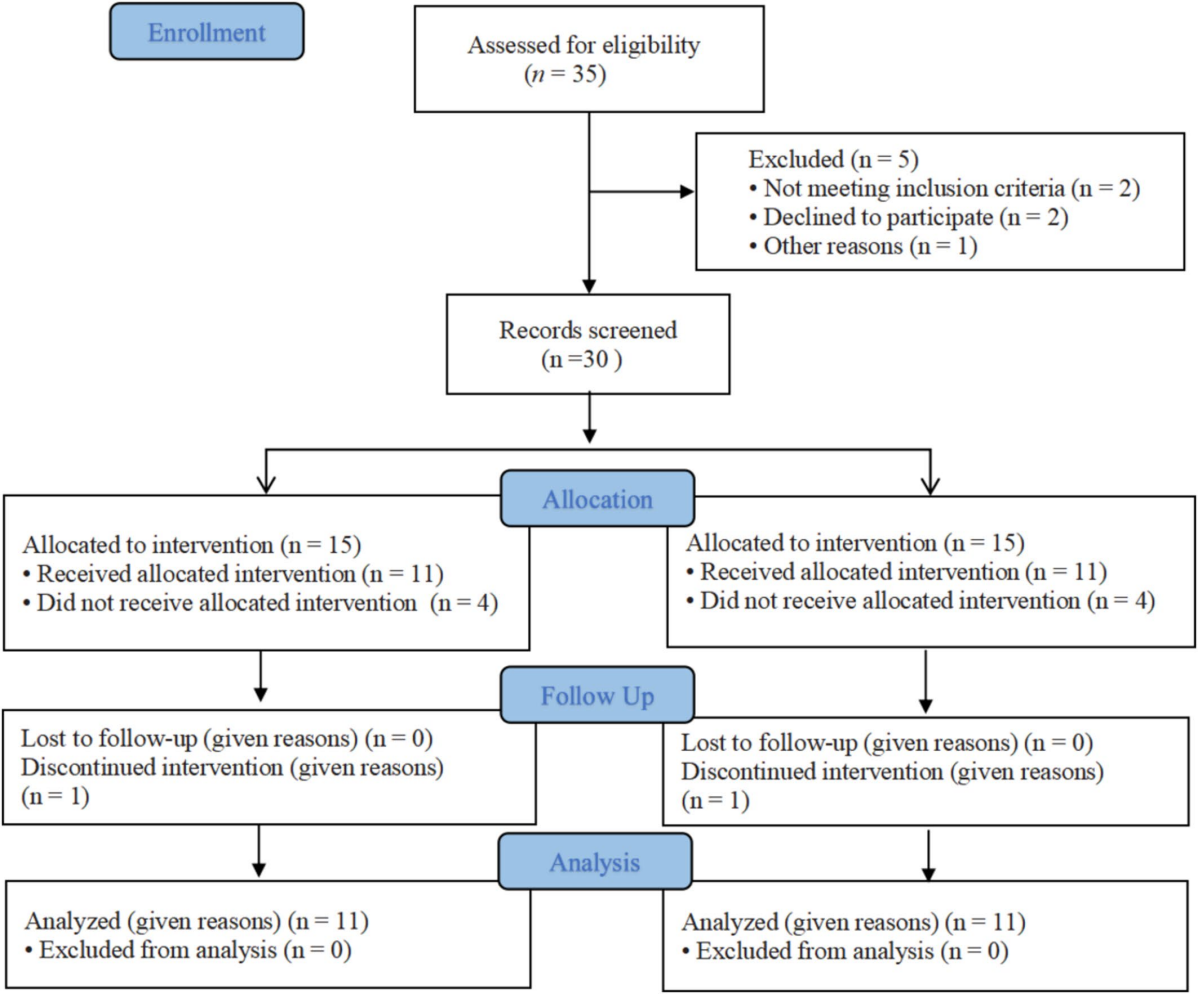
CONSORT flow graph showing the enrollment, allocation, follow-up and analysis of participants.

All patients were informed of the risks, efficacy, and precautions before treatment and signed informed consent forms. This study was approved by the Ethics Committee of Tianjin Hospital and meets the basic ethical requirements. Each participant fully understood the intervention process and expected efficacy of the trial, voluntarily participated in the trial, and signed informed consent forms before the trial. The study flow chart is shown in Figure 1.

### 2.2 Inclusion criteria

1. Met the diagnostic criteria for CPN injury as outlined in “Practical Neurology”: history of trauma; abnormal limb posture: limbs with peripheral nerve injury show varying degrees of deformity; motor impairment: muscle weakness and varying degrees of muscle atrophy; sensory impairment: disturbance in deep and superficial sensations and compound senses in the sensory distribution area.
2. Age 30–65 years.
3. Sunderland nerve classification: Grade II (axonotmesis with intact endoneurium and perineurium).
4. Hyperesthesia, electric-like numbness, pinprick sensations, and other sensory abnormalities.
5. Manifestations of CPN injury caused by pelvic fractures.
6. Willingness to participate in the trial and ability to complete related examinations.

### 2.3 Exclusion criteria

1. Unhealed fractures of the ischium and ankle.
2. Any psychological or medical condition that would affect the patient’s ability to comply with the study protocol.
3. Presence of venous thrombosis.
4. Visual-perceptual impairments such as unilateral neglect or hemianopia.
5. Moderate to severe hypertension.
6. Patients with apraxia.
7. Bed angle ≤45° (If the angle of the torso is less than 45°, MT cannot be effectively performed).
8. Cognitive or speech dysfunction.
9. Patients and their families who are uncooperative and cannot complete the trial.

### 2.4 Intervention methods

#### 2.4.1 Electric stimulation group

The NMES group utilized the KT-90A low-frequency neuromuscular electric stimulation device to conduct neuromuscular electric stimulation interventions. The patient was seated or semi-reclined (using a cradle bed). The electrode pads were placed on the tibialis anterior muscles of both legs, one on the muscle belly and one on the tendon area. NMES was applied to the affected side, while the healthy side served as a placebo, with no electrical stimulation applied. Based on previous studies, the parameters used were 2 Hz, square wave, 30-40 mA, with continuous stimulation for 30 min at the maximum intensity tolerable by the patient, aiming for noticeable muscle tremors. The intervention was performed 4 times a week, 30 min per session, for a total of 4 weeks, with intensity adjustments based on patient tolerance.

#### 2.4.2 The MT + NMES group

##### 2.4.2.1 Electric stimulation: similarly to the control group

MT: The patient was seated or semi-reclined (using a cradle bed) during MT. The affected limb was properly positioned on an adjustable support to allow flexible adjustment of the lower limb according to the patient’s physical state and activity needs. Meanwhile, the unaffected limb should maintain the same position as the affected limb to ensure both limbs presented identical postures on either side of the mirror. It is crucial to communicate with the patient, explaining the specific steps and scientific basis of MT, so the patient fully understands the background, goals, and potential side effects of the therapy. Through thorough explanations and guidance, the patient’s confidence in the treatment can be enhanced, fostering trust and cooperation. The mirror side faced the unaffected limb, while the non-mirror side faced the affected limb. Patients were required to focus on the mirror image, shifting their attention to the affected limb to increase limb awareness, and combine it with rehabilitation training movements. Under the illusion of the unaffected limb’s movement, patients were to observe and imagine the affected limb functioning normally, performing bilateral movement training independently or with assistance, repeating multiple times.

##### 2.4.2.2 Selected movements

1. Dorsiflexion: Foot moves toward the dorsum.
2. Plantarflexion: Foot moves toward the sole.
3. Eversion: Sole turns outward.
4. Inversion: Sole turns inward.

The intervention was performed 4 times a week, 30 min per session, for a total of 4 weeks, with 20 repetitions per set, and each movement repeated in 5 sets, with 25 s rest between sets.

**Figure 2:**
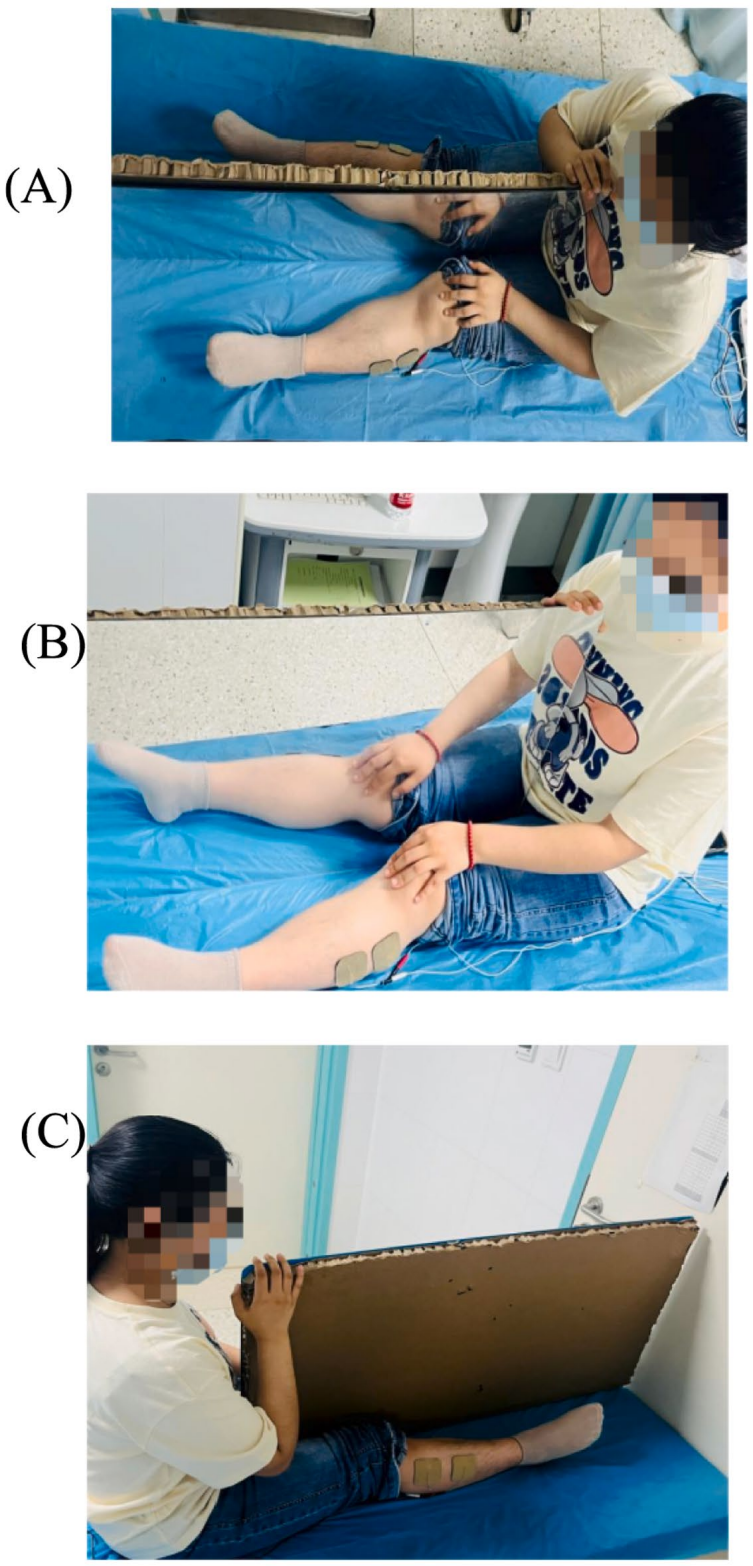
Unit group pattern diagram. **(A)** Front view; **(B)** Back view; **(C)** Top view.

Note: Adjust the intensity appropriately based on the patient’s condition without causing pain or other discomfort. If the patient cannot complete the movements, passive movements can be attempted to help the patient complete the actions, maintaining a steady pace to achieve maximum range each time.

The MT + NMES group simultaneously applied both intervention methods to the patient for a total duration of 30 min to enhance efficacy (Figure 2).

### 2.5 Testing indicators

In this study, all eligible participants were required to undergo relevant indicator testing before and 4 weeks after the treatment. To ensure the comprehensiveness and accuracy of the study, detailed basic information of all participants, including age, gender, height, weight, and medical history, was collected before the trial began. There were no significant differences in general conditions between the two groups of patients. Both the electric stimulation group and the MT + NMES group underwent surface electromyography testing, range of motion, humidity, and other indicators 4 weeks post-operation.

#### 2.5.1 Nerve conduction function

Nerve conduction measurement is an objective and quantitative examination widely used in the diagnosis of peripheral nerve injuries due to its good reliability, quantifiability, and objectivity. It can reflect the functional state of major nerve fibers, particularly sensitive to the CPN injury in the lower limbs. By studying motor conduction, the functional state of motor nerve axons, neuromuscular junctions, and muscles can be assessed. The commonly used format is electromyography, a standard method for examining nerve injuries. The testing instrument’s two electrodes are connected in series with the nerve, forming a closed circuit. As long as there is current, the nerve continuity is maintained. The trial selected the electromyograph (Nicolet EXD electromyography evoked potential system) to perform sensory conduction function and motor conduction function tests. Indicators selected include the amplitude of motor nerve action potentials, referring to the lower limb CPN injury detection standards in “The Extraction Of Neural Strategies From The Surface Emg” (Farina et al., 2024) to observe changes in values.

#### 2.5.2 Surface electromyography testing

Surface Electromyography (sEMG) is a technique that records muscle surface electrical activity to evaluate muscle function and neuromuscular control. It uses surface electrodes placed on the skin to record muscle electrical signals. The recorded signals are processed through amplification and filtering to extract various parameters reflecting muscle activity, such as root mean square (RMS) value and mean frequency.

These parameters can be used to assess muscle functional status. By analyzing and interpreting these electrical signals, information about muscle activity state and fatigue levels can be understood. Due to its non-invasive, real-time, and objective nature, sEMG is commonly used in clinical settings to monitor muscle function recovery.

The selected surface electromyograph (Tought Technology SA7550) monitored the tibialis anterior who innervated by the CPN injury. Disposable electrode pads (Ag/AgCl) were placed on the muscle belly and tendon. For the tibialis anterior muscle, electrodes were placed 1/4–1/3 of the way from the knee joint to the ankle joint, near the tibia, with a 2 cm inter-electrode distance. The movement selected was ankle plantarflexion, performed 3 times, 5 s each, with a 5 s rest interval. Using the BioNeuro Infiniti (multimedia biofeedback and data acquisition system), patient information was logged, and four-channel surface electromyography monitoring was selected. The patient relaxed for 20 s, and each movement cycle was performed in three sets. Signals and data were recorded via electrodes and transmission equipment into a computer. After recording muscle electrical activity, the data were analyzed, and the average value of the measured values during the movement cycle was selected:

1. Median Frequency (MF): The median value of the discharge frequency during muscle contraction, typically decreasing with increased exercise duration, sensitively reflecting muscle fatigue.
2. Root Mean Square (RMS): Reflects the recruitment degree of local muscle motor units and the amplitude characteristics of surface electromyography signals.

#### 2.5.3 Range of motion

Range of motion (ROM) is a straightforward and intuitive method for observing joint activity, commonly used to evaluate ankle joint function. This study selected the maximum active range of motion for the patients.

1. Dorsiflexion: The subject was supine or seated, with the knee flexed to 90°. The ankle joint was in a neutral position, free from inversion, eversion, or rotation. The goniometer’s axis was precisely placed 2.5 cm below the ankle midpoint to ensure measurement accuracy. The stationary arm aligned with the fibular shaft, while the moving arm aligned with the fifth metatarsal, measuring the angle of foot dorsiflexion.
2. Plantarflexion: The same method was used, measuring the angle of foot plantarflexion.
3. Inversion: The axis was set at the ankle joint’s center point, between the lower ends of the tibia and fibula, The stationary arm must remain parallel to the long axis of the fibula, while the moving arm aligns with the plantar surface of the foot, measuring the maximum angle of inward foot movement.
4. Eversion: The same method was used, measuring the maximum angle of outward foot movement.

During initial measurement, the angle between the stationary arm and the moving arm was 90°. After securing the position, the measurement was taken to the maximum range, and 90° was subtracted from the final reading to obtain the correct ROM. The test is conducted three times, averaging the results.

#### 2.5.4 Monofilament test

The Monofilament Test is a method used to assess tactile sensitivity, commonly used to check touch thresholds. During the test, the examiner holds a monofilament (Baseline® Fold-Up TM monofilament evaluator), pulling it out at a 90° angle. Based on the areas of numbness or reduced sensation marked on the patient, the patient is instructed to close their eyes and feel the touch. The monofilament is gently pressed against the skin (the big toe was selected; May and Morris, 2017), gradually increasing the force until the patient feels the pressure. The patient is required to report the sensation. If the patient does not feel the monofilament, a thicker one is used, and the data are recorded (Chu et al., 2023). The examiner records the necessary pressure and the patient’s sensation description. The monofilaments are categorized by thickness and specifications, with colors ranging from red, purple, blue, to green, becoming thinner and smaller. The test is conducted three times, averaging the results.

#### 2.5.5 Vibration sense

The Vibration Sense Test with a tuning fork is commonly used in neurological examinations to assess sensory system function. This method uses a tuning fork to generate vibratory stimuli on the skin surface. By observing and recording the patient’s sensation and response to the stimuli, the deep sensory function can be preliminarily judged. The patient should be relaxed, sitting or lying comfortably, and relaxing their muscles. The examiner places the vibrating tuning fork on the patient’s bony prominence (proximal joint of the big toe), using a Ryder-Seiffer tuning fork for semi-quantitative vibration sense measurement. This tuning fork, with a 128 Hz frequency, has a black and white triangular cone on its ends marked from 0 to 8. As the vibration diminishes, the triangular cone moves upwards, creating a gray triangular cone image. The patient is asked to report the vibration sensation, and the examiner notes the scale when the patient no longer feels the vibration. The test is conducted three times, averaging the results.

#### 2.5.6 Humidity

The peripheral nervous system includes the autonomic nerves, which regulate internal organs, cardiovascular functions, and gland secretion. Foot humidity is used to assess nerve recovery, an early sign of nerve injury. Sweat gland activity can be detected by evaluating sudomotor function using bioimpedance. Amy Drexeliusetal used sweat testing as a diagnostic tool for peripheral nerve degeneration diseases (DrexeliusA et al., 2022). Therefore, a humidity meter was chosen to read measurements, placed on the big toe’s tip, closely adhered to the skin for 1 min, and recorded once the value stabilized.

### 2.6 Statistical analysis

Statistical analysis was performed using SPSS 26.0 software. For normally distributed data, two-way analysis of variance (Two-way ANOVA) was used for intra-group comparisons, while Generalized Estimating Equations (GEE) were applied for non-normally distributed data. Normally distributed data were expressed as “(X ± S),” while non-normally distributed data were expressed as “median, interquartile range.” Differences with *p* < 0.05 were considered statistically significant, and those with *p* < 0.01 were considered highly statistically significant.

## 3 Results

### 3.1 General information of subjects

A total of 30 subjects were enrolled in this study, with 8 dropouts. Four subjects withdrew due to work commitments, two males were excluded due to significant data discrepancies caused by equipment malfunction, and two withdrew due to intolerable pain during electromyography collection. Ultimately, 22 patients were included. Common peroneal nerve patients (11 in the electric stimulation group and 11 in the MT + NMES group) underwent two tests, pre- and post-intervention. Two were no significant differences in gender, age, and duration of nerve injury among the two groups (*p >* 5; Table 1).

**Table 1:** Characteristics of patients with CPN injury.

	Control group (*n* = 11)	MT + NMES group (*n* = 12)	*p*-value
Sex	8(66.7%)/4(23.3%)	8(66.7%)/4(23.3%)	0.627
Age (yr)	40.6 ± 18.6	47.8 ± 15.9	0.387
The time of injury (d)	0.39 ± 0.37	0.40 ± 0.27	0.551

### 3.2 Comparison of various indicators

Following treatment, all indicators showed notable changes, and the statistical results are summarized in Table 2.Before treatment, the statistical results of the inter-group comparison showed no significant difference in the motor conduction amplitude between the Control group and the MT + NMES group (*p* > 0.05). After treatment the average motor conduction amplitude of the Control group was 0.98 ± 0.73, while the MT + NMES group had an average of 5.15 ± 3.23. Inter-group comparison showed a significant difference (*p* < 0.001). The within-group comparison after treatment indicated that the motor conduction amplitude significantly increased in the MT + NMES group (*p* < 0.01), while there was no significant difference in the Control group. Both time factors and intervention type had a significant impact on nerve conduction velocity. The MT + NMES group demonstrated superior effects in improving nerve conduction velocity compared to the NMES group, and this effect became more pronounced over time. Therefore, MT with NMES significantly improved the nerve conduction velocity in patients with CPN injury, showing better therapeutic efficacy.

**Table 2:** Summary table of statistical results.

Group	Count	df	Indicator	Mean		Within-group effect		Time effect		Inter-group effect		Interaction effect	
				Before	After	F	*p*	F	*p*	F	*p*	F	*p*
ES	11	1	Motor Conduction Amplitude	0.74 ± 1.04	0.98 ± 0.73		0.299		<0.001		0.298		<0.009
UG	11			1.65 ± 2.37	5.15 ± 3.23		<0.001				<0.001		
ES	11	1	RMS	82.71 ± 51.36	78.57 ± 67.35		0.865		0.5320		0.736		0.398
UG	11			73.39 ± 31.17	103.98 ± 81,92		0.356				0.405		
ES	11	1	Median Frequency	113.25 ± 31.38	114.12 ± 33.55	0.010	0.923	2.409	0.136	.077a	.784a	1.996	0.173
UG	11			87.54 ± 29.57	106.00 ± 18.75	4.396	0.049			8.518b	.008b		
ES	11	1	Dorsiflexion	10.36 ± 9.00	15.45 ± 10.32	3,739	0.067	5.72	0.022	.666a	.424a	0.61	0.441
UG	11			8.36 ± 6.67	18.36 ± 14.35	14,427	0.001			.093b	.764b		
ES	11	1	Plantarflexion	16.45 ± 11.45	17.45 ± 10.72	0.873	0.361	1.26	0.268	.133a	.720a	0.64	0.430
UG	11			13.60 ± 9.52	19.44 ± 8.36	30.272	<0.001			.486b	.494b		
ES	11	1	Inversion	10.17 ± 10.23	10.78 ± 8.54	0.139	0.714	2.82	0.101	1.361a	.257a	2.15	0.151
UG	11			11.13 ± 6.92	20.05 ± 11.35	29,768	<0.01			1.687b	.209b		
ES	11	1	Eversion	11.87 ± 7.10	14.76 ± 7.92	2.379	0.139	1.52	0.225	.004a	.953a	0.03	0.872
UG	11			7.45 ± 8.13	15.05 ± 11.26	16.441	<0.001			1.261b	.275b		
ES	11	1	Monofilament Test	4.61 ± 0.97	4.30 ± 0.99	1.778	0.197	27.384	<0.001	.381a	.544a	11.203	0.003
UG	11			4.61 ± 0.97	4.30 ± 0.99	36.809	<0.001			4.254b	.052b		
ES	11	1	Vibration Sensation Scores	6.59 ± 0.99	6.68 ± 0.75	0.527	0.479	25.314	<0.001	.084a	.775a	16.036	<0.01
UG	11			6.24 ± 1.04	7.04 ± 0.87	40.822	<0.001			.028b	.868b		
ES	11	1	Humidity Values	0.64 ± 0.17	0.72 ± 0.14	6.865	0.016	30.392	<0.001	.594a	.450a	3.267	0.086
UG	11			0.77 ± 0.13	0.93 ± 0.04	26.793	<0.001			7.579b	.012b		

“a” indicates the comparison between groups before treatment, while “b” indicates the comparison between groups after treatment.

Before treatment, the inter-group comparison statistical results showed no significant difference in the RMS values between the MT + NMES group and the Control group (*p* > 0.05). After treatment, the RMS assessment of the tibialis anterior muscle in the MT + NMES group significantly increased (pre-treatment: 73.39 ± 31.17, post-treatment: 103.98 ± 81.92), while the Control group showed a decrease (pre-treatment: 82.71 ± 51.36, post-treatment: 78.57 ± 67.35). Within-group comparisons showed no significant difference in the MT + NMES group (*p* > 0.05). Inter-group comparisons indicated no statistical difference in RMS values between the MT + NMES and Control groups after treatment (*p* > 0.05). The time effect was not significant, and there were no statistically significant changes in RMS values over time. The impact of time factors on RMS values was minimal.

Before treatment, the inter-group comparison statistical results showed no significant difference in the sEMG median frequency between the Control group and the MT + NMES group (*p* > 0.05). After treatment, the Control group had a median frequency of 114.12 ± 33.55, and the MT + NMES group had a median frequency of 106.00 ± 18.75, which was statistically significant (*p* < 0.05). Within-group comparisons showed a significant difference in the median frequency of the MT + NMES group (*p* < 0.05). Neither the time effect nor the interaction effect was significant.

After treatment, within-group comparisons revealed significant improvements in the ROM of dorsiflexion, plantarflexion, inversion, and eversion in the MT + NMES group (*p* < 0.01). However, inter-group comparisons showed no significant difference between the two groups for these ROM measures after treatment (*p* > 0.05). Dorsiflexion was the only joint ROM measure that showed a significant change with time, indicating that the change in dorsiflexion ROM was significant pre- and post-intervention. For the other three joint ROMs (plantarflexion, inversion, and eversion), neither time factors nor intervention type had a significant effect. The interaction effect had no significant impact on any of the four joint ROM measures.

Before and after treatment, the inter-group comparison statistical results showed no significant difference in the monofilament test between the Control group and the MT + NMES group (*p* > 0.05). Within-group comparisons after treatment indicated significant improvements in the monofilament test in both the MT + NMES group and the Control group (*p* < 0.001). The interaction effect was significant, indicating that the results of the monofilament test were significantly influenced by both time and intervention type.

Before and after treatment, the inter-group comparison statistical results showed no significant difference in the vibration sense scores between the Control group and the MT + NMES group (*p* > 0.05). Within-group comparisons showed that the vibration sense scores significantly improved in the MT + NMES group after treatment (*p* < 0.01), while there was no significant difference in the Control group (*p* > 0.05). The time effect was significant (*p* < 0.001), indicating that the time factor had a significant influence on the vibration sense scores.

**Figure 3:**
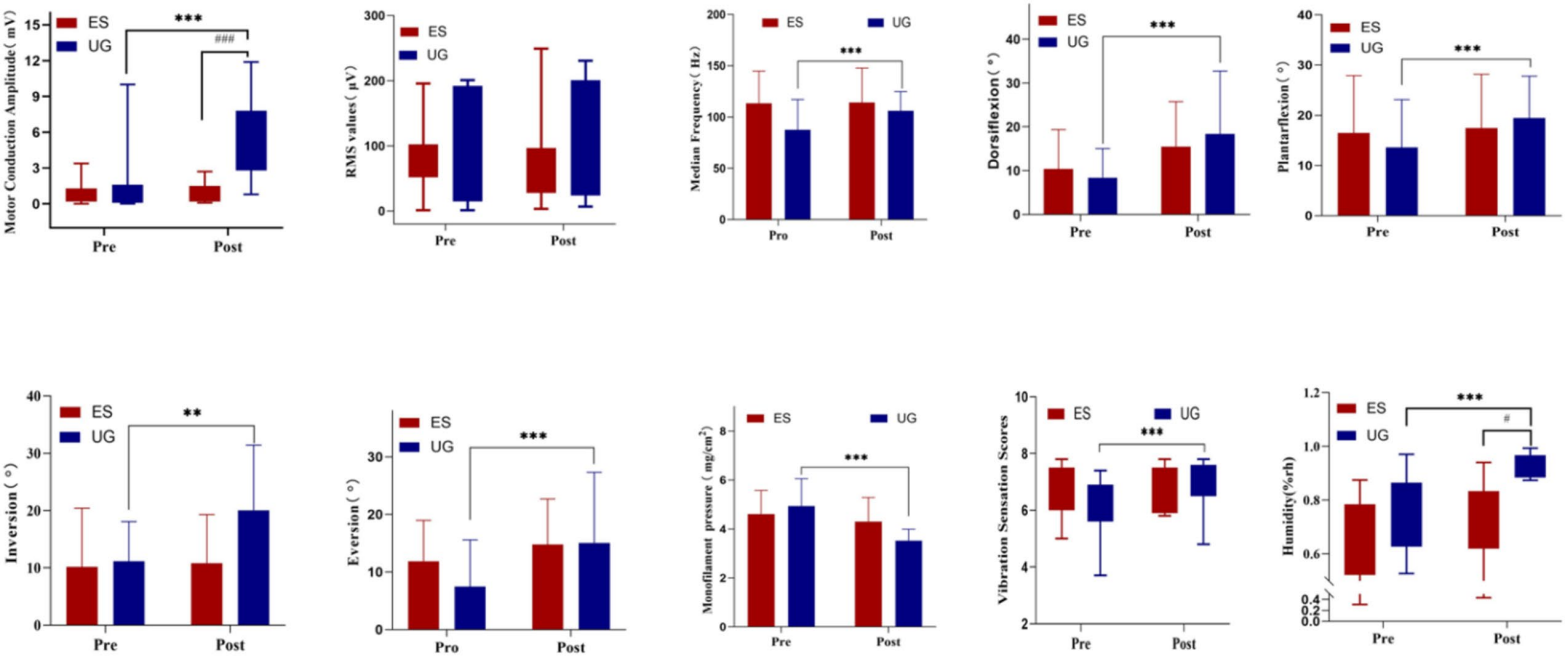
The changes in various indicators before and after treatment *Intra-group comparison, *p* < 0.05; Intra-group comparison, *p* < 0.01; *Intra-group comparison, *p* < 0.001; #Inter-group comparison, *p* < 0.05; ##Inter-group comparison, *p* < 0.01; ###Inter-group comparison, *p* < 0.001.

Before treatment, the inter-group comparison statistical results showed no significant difference in the skin humidity values between the Control group and the MT + NMES group (*p* > 0.05). After treatment, the MT + NMES group showed a significant difference in skin humidity values (*p* < 0.05). Within-group comparisons showed significant improvements in skin humidity in the MT + NMES group (*p* < 0.001), while there was no significant difference in the Control group (*p* > 0.05). The time effect was significant, while the interaction effect was not. The intervention significantly improved the humidity values, indicating that NMES United with MT improved the skin humidity in patients with CPN injury. Figure 3 illustrates the specific changes in various indicators before and after treatment.

## 4 Discussion

The results of this study indicate that the NMES group and MT + NMES group demonstrated a certain therapeutic effect on functional recovery after Common peroneal nerve. Specifically, for humidity values, nerve evoked potentials, and monofilament test indicators, the MT + NMES group showed better effects than the NMES.

RMS represents the effective value of nerve discharge by calculating the root mean square value of all amplitudes in an instantaneous electromyogram over a specific period. It is used to express the number of motor units activated during muscle activity. Kim et al. (2011) found that RMS could also be used for limb motor function testing and functional evaluation to reflect muscle strength levels. Evaluating muscle strength using surface EMG, compared to needle EMG, offers a safe, painless, and non-invasive approach, allowing digital recordings to assess the average force level of specific muscle activation (Munoz-Novoa et al., 2022). In this study, after 4 weeks of treatment, the RMS value of the tibialis anterior muscle increased in the MT + NMES group compared to before treatment, while the RMS value decreased in the NMES group. Inter-group comparisons between the MT + NMES group and the NMES group also showed no significant differences. These findings suggest that neither the NMES group nor the combined therapy group significantly altered the RMS values in this study. Mu et al. (2007) found that NMES mimicking the action potential pattern of slow muscle fibers induced a shift from fast to slow fiber types in denervated muscles, primarily through the calcium/calmodulin-dependent kinase (CaMK) pathway, promoting a transition from strength-oriented to endurance-oriented muscle characteristics, leading to increased endurance but decreased muscle strength. In this study, no significant differences were found between the tibialis anterior muscle groups. It is speculated that the tibialis anterior muscle mainly comprises slow muscle fibers, nerve injury affects fast muscle fibers more significantly, which may explain the observed phenomena in this experiment.

Median frequency is widely used clinically to evaluate muscle disorders and analyze muscle fatigue. A higher median frequency indicates a higher level of muscle fatigue resistance, relatively higher muscle endurance (Xu et al., 2024). In this study, after 4 weeks of treatment, the median frequency evaluations of the tibialis anterior muscle improved in the NMES group and MT + NMES group compared to before treatment, There was a significant difference between the groups. It is hypothesized that NMES may stimulate the muscles through neuromuscular electrical activity, while MT could activate brain regions associated with neural repair via visual feedback, potentially enhancing the brain’s ability to adapt to the damaged nerves. These two therapeutic approaches may interact, leading to a more pronounced treatment effect compared to NMES alone, a significant difference between the groups. It is hypothesized that NMES may stimulate the muscles through neuromuscular electrical activity, while MT could activate brain regions associated with neural repair via visual feedback, potentially enhancing the brain’s ability to adapt to the damaged nerves. MT designs exercise movements based on the number of repetitions, frequency, and duration completed by the patient. The intensity gradually increases as the patient progresses, thereby enhancing muscle strength and improving the ability to perform the activities. These two therapeutic approaches may interact, leading to a more pronounced treatment effect compared to NMES alone.

Injury to the common peroneal nerve can lead to paralysis of the extensor muscles on the anterior and lateral sides of the leg, limiting dorsiflexion and eversion angles, affecting patients’ walking and mobility (Garozzo et al., 2004). In this study, after 4 weeks of treatment, all groups showed improved ranges of motion for dorsiflexion, plantar flexion, inversion, and eversion compared to before treatment. However, no significant differences were observed within the NMES group. In contrast, significant differences were found within the MT + NMES group, but no significant differences were observed between the groups. Therefore, MT with NMES can expand the lower limb joint range of motion in patients with Common peroneal nerve. After nerve injury, denervation occurs, causing muscle atrophy and inability to facilitate normal movement, making joint range of motion a relatively intuitive reflection of nerve recovery. In this study, we found that patients in the NMES group showed no significant improvement. NMES provides intermittent stimulation, generating passive motion conduction without establishing proper motor memory and action patterns in the brain, leading to motor abilities not matching the movement patterns, ultimately resulting in insignificant therapeutic effects. In contrast, the MT + NMES group incorporated MT, which activates rigid motor patterns through three aspects. First, utilizing the continuous visual feedback mechanism effectively stimulates the primary motor cortex (M1) of the human brain. This stimulation significantly affects the brain’s electrophysiological activity and excitability levels, promoting brain function remodeling. This mechanism induces and facilitates the recovery of motor function. Secondly, patients conduct bilateral motor training independently or with assistance, involving ankle joint movements such as dorsiflexion, plantar flexion, eversion, and inversion. These movements activate the motor cortex extensively through signals transmitted via the spinothalamic tract, facilitating partial motor pathways on the affected side and promoting limb motor function recovery. Thirdly, under the “healed” incorrect image stimulation of the affected limb by MT, repeated training reduces learned non-use and promotes motor function recovery. No significant differences were found between the groups, possibly because generating lower limb activities requires recruiting a large number of motor units, forming a complete motor pattern in the brain, and considering factors such as fractures, swelling, and stiffness that accompany patients. Even when the activity conditions are met, no significant motor performance is achieved.

Feng et al. (2009) found that examining three sites on the plantar surface, namely the great toe, third metatarsal, and fifth metatarsal, maximizes the diagnostic value of the monofilament. The smaller the monofilament size, the lower the pressure, indicating greater skin sensitivity and more significant therapeutic effect. In this study, after 4 weeks of treatment, the pressure threshold measured by monofilament testing decreased in all patients compared to before treatment. The improvement in the Control group was not statistically significant, whereas the improvement in the combined group was significant. The sensory recovery sequence following nerve injury typically begins with the restoration of pain and temperature sensations controlled by myelinated and unmyelinated fibers, and subsequently vibratory sensation, culminating in discriminative sensation. Touch-related superficial sensations recover faster than vibratory sensations. Peripherally, NMES can promote sensory input from tactile corpuscles, enhancing nerve regeneration and improving sensory function. Elabd et al. found that only about 40% of regenerated axons of sensory neurons could re-enter the original branches after sensory nerve transection, but low-frequency NMES could increase this rate to around 75% (Elabd et al., 2022) accelerating nerve growth and sensory function recovery. At the central level, superficial sensory conduction pathways primarily handle the transmission of pain, temperature, crude touch, and pressure sensations from the skin and mucosa. These sensory impulses ascend along nerve fibers, first reaching the spinal nerve root ganglion for the first-order neuron relay. Subsequently, the impulses continue along nerve fibers to the posterior horn of the spinal cord for the second-order neuron relay. At this stage, the fibers cross to the opposite side and ascend further, eventually reaching the thalamus, completing the third-order neuron relay. At the thalamus, nerve fibers project further to the postcentral gyrus of the cerebral cortex, achieving sensory integration and recognition. Any pathological changes along this pathway can result in varying sensory impairments. Zhang et al. (2018) found that mirror visual feedback can activate the ipsilateral primary motor cortex and the mirror neuron system in individuals with stroke. Long-term motor execution using mirror visual feedback can induce the transfer of activation to the ipsilateral hemisphere, where motor-related regions are activated, thereby enhancing neural activity in the mirror neuron system of the affected hemisphere. This promotes the reorganization and repair of the motor-related cortex in the brain. MT can enhance neural activity in relevant brain regions through dual stimulation of vision and motion, optimizing superficial sensory pathway conduction and improving neural conduction efficiency, thus enhancing superficial sensation.

This study found that after 4 weeks of treatment, vibration sensation test values improved in all groups, with the MT + NMES group showing significant improvement pre- and post-treatment but no significant intergroup differences. Therefore, MT with NMES can improve vibratory sensation in patients with Common peroneal nerve. The sensory recovery sequence follows: pain and temperature sensation-touch-vibratory sensation-discriminative sensation. The intrinsic law of motor function recovery follows a precise and orderly sequence of steps. Initially, sensory input involves external force assistance to facilitate perception recovery and establishment. Subsequently, proprioceptive input gradually dominates, enabling individuals to rely on their sensory systems for motor regulation without external force assistance. Through repeated training and practice, motor patterns are standardized and fixed, a crucial step in motor function recovery. Subsequently, multiple or excessive repetitions of standard movements are vital for consolidating and strengthening motor patterns, helping establish corresponding motor function areas in the cerebral cortex. Ultimately, as these steps progress, motor function is regained, enabling individuals to execute complex motor tasks anew. For motor reconstruction, appropriate proprioceptive input is essential. Weiller found that afferent signals integrated by the central nervous system (Weiller et al., 1993) transmit motor control signals via the corticospinal tract to motor organs, enhancing cortical excitability and further improving the execution of related movements post-nerve injury. The lack of significant differences between groups can be attributed to two factors: firstly, deep sensory receptors are primarily distributed in muscles, tendons, joints, and ligaments, and NMES acts superficially, insufficiently affecting deep sensory receptors, hindering deep sensory input and perception. Secondly, deep sensory recovery lags behind superficial sensory recovery, resulting in less pronounced effects on deep sensation under the same treatment conditions. Additionally, the body’s proprioception is obtained through muscle contraction or relaxation, as well as the stretching of tendons and ligaments. Patients with limited joint range of motion may not adequately perform full-range joint movements, diminishing proprioceptive input and impacting therapeutic efficacy.

EMG is an objective quantitative examination widely used in diagnosing peripheral nerve injuries due to its reliability, quantifiability, and objectivity. Nerve conduction velocity, recognized as the “gold standard,” is extensively acknowledged by scholars both domestically and internationally. By detecting nerve conduction velocity, the actual location of nerve injury can be identified, and nerve regeneration status can be clarified. In this study, after 4 weeks of treatment, nerve evoked potentials improved in both groups compared to before treatment, with the MT + NMES group significantly outperforming the NMES group, showing significant differences between groups after treatment. This indicates that the MT + NMES group can significantly enhance nerve evoked potentials following Common peroneal nerve, accelerating the nerve regeneration process. Sasha et al. discovered that for myelinated large nerve fibers such as the common peroneal nerve and sciatic nerve (Smith et al., 2024), nerve conduction velocity is more precise and objective. After nerve injury, demyelination leads to significant changes, and metabolic biochemical abnormalities occur in nerve tissue proteins, weakening the regenerative capacity of nerve fibers, resulting in significantly reduced conduction velocity and action potential amplitude (Gao et al., 2020). Therefore, increased amplitude indicates more regenerated nerve axons, implying improved nerve regeneration. EMG can represent nerve conduction velocity, indirectly reflecting nerve regeneration through changes in amplitude values. Senger et al. found that 20 Hz low-frequency NMES could increase the number and length of axons per unit time, promoting nerve regeneration (Senger et al., 2018). Taylor et al. found that after complete transection of the upper limb peripheral nerve and microsurgical repair, the right anterior insula exhibited significant cortical thinning and gray matter reduction, affecting the body’s homeostatic input (Taylor et al., 2009). The study results show that RMS, monofilament tests, and other indicators demonstrate significant improvement in motor and sensory functions after nerve regeneration. The closed-loop rehabilitation theory cleverly combines “central intervention” and “peripheral intervention,” providing positive feedback through the synergistic effects of central and peripheral interventions to achieve continuous optimization of therapeutic effects (Chen et al., 2020; Remsika et al., 2021). In the closed-loop rehabilitation model, NMES activates peripheral proprioceptors, mimicking sensory external feedback activation. Signals are transmitted to the central nervous system via the spinothalamic tract. Early underactivity, NMES can lower the excitability threshold of the motor cortex, making limb movements more likely to occur. MT, through imagination, execution, and observation, activates relevant brain regions, enhancing synaptic experience-dependent plasticity and neural plasticity, stimulating neurotrophic and nerve growth factors related to neural plasticity, further improving central programming, and activating and regulating the central nervous system to promote neuronal regeneration and connectivity. This combined approach utilizes the advantages of central and peripheral interventions to treat peripheral nerve injuries effectively.

This study found that after 4 weeks of treatment, vibration sensation test values improved in two groups, with the MT + NMES group showing significant improvement pre- and post-treatment and with a significant intergroup differences. This indicates that the MT + NMES group can significantly improve sudomotor humidity values following Common peroneal nerve. Postganglionic peripheral neuropathy can cause autonomic symptoms, manifested as sudomotor dysfunction due to reduced epidermal nerve innervation and the presence of degenerative nerve fibers in the dermis, leading to small fiber sensory neuropathy and affecting activities (Tronstad et al., 2013). The brain includes the limbic system and neocortex, which regulate visceral activities, with the amygdala in the limbic system indirectly influencing sudomotor activity and the neocortex area 6 closely related to sweating and vascular vasomotion in the limbs (Oh et al., 2023). MT, based on mirror neurons, shares overlapping regions with the limbic system and cerebral cortex, suggesting that activating mirror neurons may improve corresponding visceral nerve function. However, no related studies have been found in the literature. Based on experimental results, it is speculated that the combined application utilizes NMES to directly and indirectly stimulate sweat gland cells, promoting sweat secretion. MT elicits various neural reflexes through exercise, acting on the cerebral cortex, activating mirror neurons, affecting neural excitability, and regulating visceral activities, making the therapeutic effect more pronounced.

## 5 Conclusion

In conclusion, MT with NMES can significantly improve surface EMG, joint range of motion, spontaneous pain levels, monofilament sensitivity tests and skin humidity in patients with CPN injury. We found significant improvements in patients’ sensory and motor functions. However, the NMES group alone also showed a certain degree of improvement. Most of the data in this study did not show a significant advantage of the combined group over the NMES group, suggesting that neuromuscular NMES is beneficial for the recovery of motor and sensory functions after common peroneal nerve injury. The combination of MT further enhances these benefits. It is recommended that both treatments be used in combination in clinical.

## Data Availability

The raw data supporting the conclusions of this article will be made available by the authors, without undue reservation.
